# Single-cell multiomics revealed the dynamics of antigen presentation, immune response and T cell activation in the COVID-19 positive and recovered individuals

**DOI:** 10.3389/fimmu.2022.1034159

**Published:** 2022-12-02

**Authors:** Partha Chattopadhyay, Kriti Khare, Manish Kumar, Pallavi Mishra, Alok Anand, Ranjeet Maurya, Rohit Gupta, Shweta Sahni, Ayushi Gupta, Saruchi Wadhwa, Aanchal Yadav, Priti Devi, Kishore Tardalkar, Meghnad Joshi, Tavpritesh Sethi, Rajesh Pandey

**Affiliations:** ^1^ Division of Immunology and Infectious Disease Biology, INtegrative GENomics of HOst-PathogEn (INGEN-HOPE) laboratory, CSIR-Institute of Genomics and Integrative Biology (CSIR-IGIB), Delhi, India; ^2^ Academy of Scientific and Innovative Research (AcSIR), Ghaziabad, India; ^3^ CSIR-Institute of Genomics and Integrative Biology (CSIR-IGIB), Delhi, India; ^4^ Indraprastha Institute of Information Technology Delhi, New Delhi, India; ^5^ Department of Stem Cells and Regenerative Medicine, Dr. D. Y. Patil Medical College, Hospital and Research Institute, Kolhapur, Maharashtra, India

**Keywords:** COVID-19, single cell multi-omics, recovered COVID-19 individuals, immune response, bayesian network model, T-cell activation

## Abstract

**Introduction:**

Despite numerous efforts to describe COVID-19's immunological landscape, there is still a gap in our understanding of the virus's infections after-effects, especially in the recovered patients. This would be important to understand as we now have huge number of global populations infected by the SARS-CoV-2 as well as variables inclusive of VOCs, reinfections, and vaccination breakthroughs. Furthermore, single-cell transcriptome alone is often insufficient to understand the complex human host immune landscape underlying differential disease severity and clinical outcome.

**Methods:**

By combining single-cell multi-omics (Whole Transcriptome Analysis plus Antibody-seq) and machine learning-based analysis, we aim to better understand the functional aspects of cellular and immunological heterogeneity in the COVID-19 positive, recovered and the healthy individuals.

**Results:**

Based on single-cell transcriptome and surface marker study of 163,197 cells (124,726 cells after data QC) from the 33 individuals (healthy=4, COVID-19 positive=16, and COVID-19 recovered=13), we observed a reduced MHC Class-I-mediated antigen presentation and dysregulated MHC Class-II-mediated antigen presentation in the COVID-19 patients, with restoration of the process in the recovered individuals. B-cell maturation process was also impaired in the positive and the recovered individuals. Importantly, we discovered that a subset of the naive T-cells from the healthy individuals were absent from the recovered individuals, suggesting a post-infection inflammatory stage. Both COVID-19 positive patients and the recovered individuals exhibited a CD40-CD40LG-mediated inflammatory response in the monocytes and T-cell subsets. T-cells, NK-cells, and monocyte-mediated elevation of immunological, stress and antiviral responses were also seen in the COVID-19 positive and the recovered individuals, along with an abnormal T-cell activation, inflammatory response, and faster cellular transition of T cell subtypes in the COVID-19 patients. Importantly, above immune findings were used for a Bayesian network model, which significantly revealed *FOS, CXCL8, IL1β, CST3, PSAP, CD45* and *CD74* as COVID-19 severity predictors.

**Discussion:**

In conclusion, COVID-19 recovered individuals exhibited a hyper-activated inflammatory response with the loss of B cell maturation, suggesting an impeded post-infection stage, necessitating further research to delineate the dynamic immune response associated with the COVID-19. To our knowledge this is first multi-omic study trying to understand the differential and dynamic immune response underlying the sample subtypes.

## Introduction

COVID-19 pandemic is into its 3^rd^ year in continuum, and it continues to pose threat to lives, healthcare support, medical infrastructure and livelihoods. This has been compounded by new emerging SARS-CoV-2 variants of concern (VOC) with differential geographical origin and emphasizing the constant need for effective vaccines/dosage. Due to the lack of antibody neutralization along with immune evasion, these emerging variants of SARS-CoV-2 have posed a global threat to human health compounded by the diversity of disease severity symptoms. Therefore, it is important to undertake studies in different population cohorts, especially high population density regions, to understand and elucidate the differential immune response. This will enable mechanistic understanding of the hosts’ immune response during COVID-19 disease. Towards this, studying the whole transcriptome along with surface marker expression at the single cell resolution can provide detailed functional insights into the COVID-19 pathology.

Previous evidence suggests that a significant immune dysregulation occurs in severe COVID-19 patients (Su, 2020). In particular, studies on peripheral blood mononuclear cells (PBMCs) have revealed reduced IFN-gamma production (1), expansion of highly cytotoxic effector T cell subsets (2), and increased expression of the exhaustion markers programmed cell death protein 1 and Tim-3 on CD8+ T cells in the severe COVID-19 patients (3). Another study revealed classical monocytes, along with monocyte chemoattractant CCL2 and its receptor CCR2, the neutrophil chemoattractant CXCL8, and TNF-α as the main mechanism of cytokine storm observed in the COVID-19 (4). A significant decrease in the non-classical monocyte genes (*C1AQ, C1BQ*, and *LSTB1* expression) and a corresponding relative increase of classical monocytes genes (*S100A8, S100A9*, and *S100A12* expression) in the critical COVID-19 patients was also observed (5). Upregulation of *IL6R* and *IL6ST* in the COVID-19 patients have been reported, which synergistically promotes increased proinflammatory cytokines during pathogenesis. Several interferon (IFN)-stimulated genes (ISGs; including *ISG15, IFI44, IFI44L*, and *RSAD2*) were also specifically upregulated in the PBMCs from the COVID-19 patients, enhancing antiviral and immune modulatory functions (6). Presence of lymphopenia, immune cell exhaustion, and elevated serum pro-inflammatory cytokines are some of the striking features of COVID-19 disease severity (1, 7, 8). Also, impaired activation of B cell subsets provides evidence to explain the delayed viral clearance in the severely ill COVID-19 patients (9). While several studies have provided a comprehensive atlas of the immune response dynamics in the COVID-19 patients, a few studies have recently highlighted the immune repertoire of the recovered individuals. In one study, neutrophil activation and migration associated genes were reported to be downregulated in the recovered individuals compared to the active infection (10). The monocyte mediated immune response was reported to be restored to normal in the convalescent COVID-19 patients (11). Another study reported a decreased T cell differentiation in the recovered individuals post severe COVID-19, but an opposite pattern post mild/moderate COVID-19 (12).

Above studies have augmented our understanding of the spectrum of immune response in the COVID-19 patients globally, except India. However, it is also true that most of the studies have reported the immune profile between the healthy and SARS-CoV-2 infected individuals. We think it would also be fruitful to understand and investigate the pathophysiology that drives the SARS-CoV-2 infection by single cell based immune profile post-infection, in the *recovered patients along with the healthy and active infection*. Along with this, in addition to the transcriptomic response, the cell surface markers can also provide vital clues about the pathophysiology, as they not only are cell-type identifiers, but they also represent the cell state as well. Yet, a comprehensive investigation of COVID-19 pathophysiology, across active COVID-19 and recovered patients, including transcriptome as well as cell surface marker is lacking. Here, for the first time, we performed simultaneous single-cell transcriptomics and single-cell Ab-seq across the healthy, COVID-19 positive and recovered individuals.

Our study reports a decreased MHC Class I-mediated antigen presentation as well as dysfunctional MHC Class II-mediated antigen presentation in the COVID-19 positive patients, followed by a restoration of the function in the recovered individuals. We also found a loss of B cell maturation process, reduced cytotoxicity and antibody response in the recovered individuals. Besides, we identified a CD40-CD40 ligand interaction-mediated increased inflammatory, immune and stress response by the monocyte, NK cells, CD4+ TCM, and CD8+ T cell populations in the COVID-19 patients. We observed a faster cellular transition within the T cell subtypes in COVID-19 patients, with a T cell-mediated perturbation of normal cellular functions alongside the immune/inflammatory response. Finally, using a Bayesian Network model, we identified *FOS, CXCL8, IL1β, CST3, PSAP, CD45* and *CD74* as predictors of the COVID-19 disease. The findings will augment the understanding vis-a-vis the heterogeneity and complexity of the immune response in the COVID-19 positive and recovered individuals, as well as provide an integrated multi-omics model for immune response during infection, post-infection and without infection.

## Methods and materials

### Patient cohort, sampling and data collection

#### Sample collection

The samples were collected at a tertiary care center (Dr. D. Y. Patil Medical College, Hospital and Research Institute, Kolhapur, Maharashtra, India) from the healthy volunteers, patients with confirmed COVID-19 positive status and patients recovered from COVID-19 (within 4 weeks) based on qRT-PCR results under the ethics oversight of the institution. The samples were matched with respect to age and gender, and the infecting SARS-CoV-2 variant was identified using Oxford Nanopore sequencing. In the COVID-19 positive group, 13 out of 16 individuals were infected with 20B, and the rest three with 20A variant. In the recovered group, 11 out of 13 were infected with 20B and rest two with 20A. The COVID-19 positive and recovered individuals were matched with respect to the disease severity. The blood samples were collected in the BD Vacutainer^®^ CPT™ Cell Preparation Tube with sodium citrate. Peripheral blood mononuclear cells (PBMC) were isolated from whole blood using the manufacturer’s recommendation (ref no 362761). The PBMCs were cryopreserved in a cryopreservation media (FBS and DMSO at 9:1 ratio) till further use.

#### Sample processing and library preparation

The PBMCs were revived and processed using BD Rhapsody single cell analysis system as per Domenico et al. (13). Briefly, 0.2 million cells per sample were taken and labelled using BD™ Single-Cell Multiplexing Kit-Human and 40 BD™ AbSeq Ab-Oligos as per manufacturer’s guide (Doc ID: 214419 Rev. 2.0). An average of 30000 pooled cells were loaded in each cartridge on the BD Rhapsody express single cell analysis system for single cell capture followed by the cDNA synthesis as per manufacturer’s guideline (Doc ID: 210967 Rev. 1.0). mRNA Whole Transcriptome Analysis (WTA), Ab-Seq, and Sample Tag library were prepared using BD Rhapsody™ WTA Amplification kit as per manufacturer’s guideline (Doc ID: 23-21752-00). The libraries were sequenced using NovaSeq 6000 S2 reagent kit at 30000 reads/cell for WTA, 20000 reads/cell for AbSeq, and 120 reads/cell/Sample Tag for sample tag library, with 101 x 2 cycles.

### scRNA-seq data processing, clustering and cell-type annotation

The raw sequencing data was demultiplexed and converted to FASTQ format using the bcl2fastq tool. The data was analyzed using BD Rhapsody WTA analysis pipeline as per manufacturer’s guideline (Doc ID: 47383 Rev. 9.0). The count matrix with recursive substitution error correction was imported to Seurat R package for downstream analysis and visualization (14). The WTA and Ab-Seq count matrices for all the healthy, active COVID-19 and recovered patients (a total of 163197 cells) were merged for integrated multimodal analysis. Quality parameters were optimized and cells containing >2500 UMI and <20UMI were discarded. Batch effects were normalized and data was normalized using Seurat SCTransform V2 (15, 16). Finally, cells were clustered using unsupervised clustering at a resolution of 0.4 and visualized with UMAP algorithm (17). Cluster specific genes were identified using FindAllMarker function (Wilcoxon rank sum test, Log2 Fold Change cut-off 1.5). Clusters were comprehensively annotated manually using a combination of CellMarker DB, PanglaoDB, and Azimuth, as well as automatically using scpred, an SVM-based single cell annotation tool. The same pipeline was also applied for a pairwise visualization of the data (healthy vs COVID-19, COVID-19 vs recovered, and healthy vs recovered).

### Differential gene expression and gene set enrichment analysis

Differential gene expression analysis was performed on clustered pairwise data (healthy/COVID-19, COVID-19/recovered and healthy/recovered) using Seurat FindMarker function (Wilcoxon rank sum test, Log2FC cut-off 1.5, q value cut-off 0.05). For cluster-wise pseudo-bulk differential gene expression analysis, average gene expression was taken at the sample-level, followed by differential gene expression analysis using DESeq2 r package across the three groups (18). Wald test was applied for the analysis, followed by the visualization using pheatmap R package. Gene set enrichment analysis (GSEA) was performed at the single cell resolution, keeping the cluster and group identity, using the escape R package. Gene set collections were used from built-in Molecular Signature Database to perform the enrichment. The relative enrichment scores for each pathway were represented in the form of a heatmap using dittoSeq R package (19).

### Signaling networks interaction inference

The Signaling network analysis was performed using CellDesigner to infer the protein and pathways interaction networks for significantly differentially expressed gene sets. The P values were corrected using the Bonferroni Method.

### Single cell pseudotime trajectory analysis

At first, the T cell subtypes were extracted from the Seurat object containing all the 17 cell types. The data was preprocessed as documented by Packer et al. (20). A principal trajectory graph was built within each partition using the learn_graph function in Monocle3 (21). The cells were ordered along with the pseudotime trajectory which follows the shortest path. Naive CD4+/CD8+ T cell was defined as the root node while constructing the trajectory. To find the modules of co-expressed genes, we used the differentially expressed genes across the healthy, COVID-19 positive and recovered individuals (pseudobulk differential expression analysis). We extracted the genes falling in each module and performed GO enrichment using Enrichr (22). GO molecular functions with statistically significant enrichment score (p value < 0.05) were selected and visualized using the ggplot2 r package.

### Multi-omics analysis

Multi- omics analysis was performed on the transcriptome, surface marker expression and clinical information. In order to avoid possible bias induced by the unequal number of cells in each sample, three samples from each group i.e., COVID-19 positive, healthy and recovered were sampled to select an equal number of cells from each sample. This stratified sampling approach resulted in 24633 cells from nine patients. Similarity network fusion was used to fuse the multi-omic data into a single matrix of size, 24633 by 24633. tSNE was performed upon the fused data to visualize seven clusters selected by the elbow method using k-means clustering. The clusters were identified manually as described earlier in the “scRNA-seq Data Processing, Clustering and Cell-Type Annotation” section.

### Clinical features and an integrated Bayesian network model

The data from stratified random samples of single cell RNA expression, surface marker and SNF cluster membership were integrated with high-resolution CT (HRCT) scores of the COVID-19 patients. The healthy and recovered individuals were assigned an HRCT score of zero, indicating absence of active pneumonia. The integrative modeling analysis was carried out using the wiseR (23) package for end-to-end Bayesian network learning, inference and dashboard deployment. All continuous variables in the integrated data were discretized using the k-means algorithm with k=3 for biological interpretability as low, medium and high. A discrete Bayesian network was learnt from the data using hill climbing optimization for finding the directed acyclic graph encoding the structural dependencies between the variables. Eleven Bayesian network structures were ensembled and averaged to derive the consensus structure. The consensus structure was then parametrized with marginal and conditional probability distributions using Monte Carlo Markov Chain (MCMC) approximate inference method.

## Results

### Spectrum of cellular heterogeneity in healthy, COVID-19 and recovered patients

**Figure 1:**
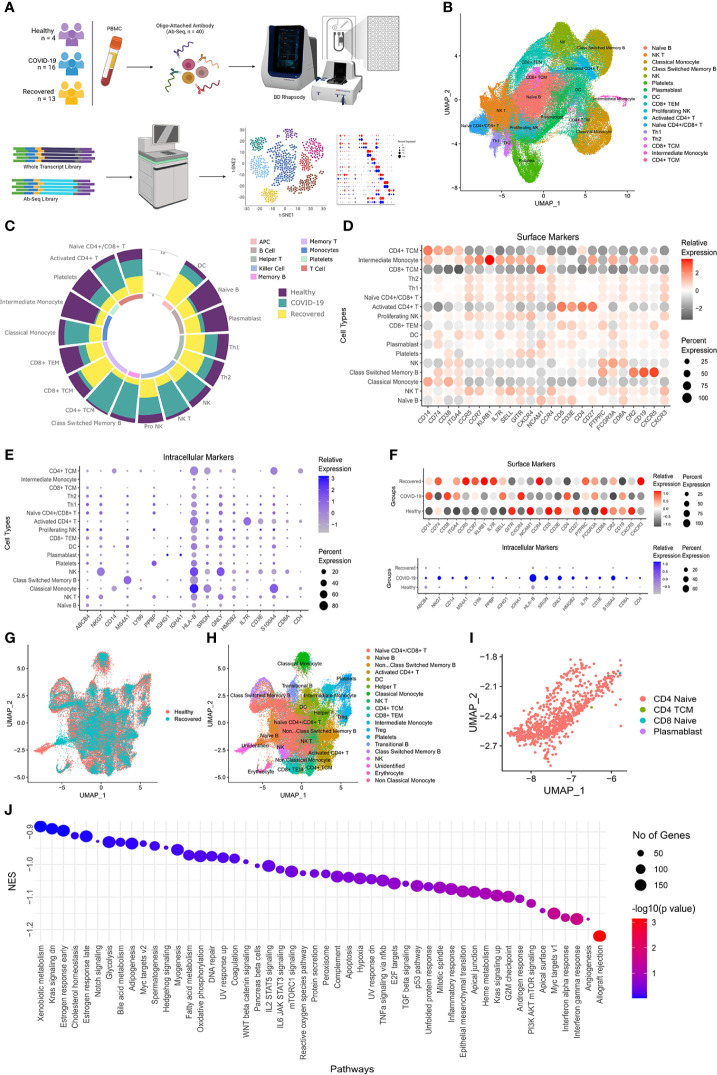
Cellular Heterogeneity across Healthy, Infected and Recovered COVID-19 Individuals. **(A)** Sample distribution and schematic workflow for the scRNA-seq, followed by analysis for the cellular heterogeneity and differential expression. **(B)** UMAP visualization of the 124726 cells across the healthy, active COVID-19 and the recovered individuals. **(C)** Frequency of cell types across the three groups, normalized to the total number of cells. **(D, E)** Cell type specific expression of **(D)** Surface markers, and **(E)** RNA level. Color scale denotes the relative expression whereas circle size denotes the percent of cells expressing the marker. **(F)** Expression of cell type specific markers across the three groups. **(G)** UMAP visualization of healthy vs recovered comparison group showing no batch effect between the two groups, the box highlights the cluster absent in the recovered individuals. **(H)** UMAP visualization of healthy vs recovered comparison group with cell type annotation, the box highlights the unidentified cluster. **(I)** UMAP visualization of the unidentified cluster after machine learning-based cell type annotation. **(J)** GSEA of differentially expressed genes between novel subset of the Naive CD4+ T cell and existing Naive CD4+/CD8+ T cells.

To understand the spectrum of immune response and cellular heterogeneity before, during and after the SARS-CoV-2 infection, we performed simultaneous single-cell transcriptome and targeted proteome (40 surface markers) sequencing of the PBMCs collected from 33 individuals (healthy = 4, COVID-19 = 16, and recovered = 13) using a microwell-based scRNA-seq platform (BD Rhapsody Express). We sequenced a total of 163197 cells for both the surface proteins and RNA (**Figure 1A**). The details of the oligo-attached antibodies (list and oligo sequences) are available in the **Supplementary File 1**; **Table S1**. Post QC of the data quality (**Supplementary File 1**; **Figure S1A**), a total of 124726 cells were retained after removing the low-quality cells. We performed batch effect correction and normalization using Seurat SCTransform v2 (24), followed by dimension reduction and unsupervised clustering using the Louvain algorithm (resolution = 0.4). The cluster specific genes were identified using Seurat FindMarker functions and the clusters were annotated manually using CellMarker, PanglaoDB and Azimuth, as well as using a Support Vector Machine (SVM)-based annotation tool scPred (the ROC, sensitivity and specificity are available at **Supplementary File 1**; **Table S2**) (14, 16, 25–27). A total of 17 annotated clusters were identified across the three groups (**Figure 1B**). **Figure 1C** represents the frequency of the cell types between the three groups (normalized to the total number of cells in the group).

### Decreased professional antigen presenting cells in the COVID-19 patients

Antigens undergo a proteolytic cleavage, followed by presentation to the CD8+ T cells and CD4+ T cells by MHC class I or class II molecule, respectively. B cells, macrophages and the dendritic Cells (DC) are the professional antigen presenting cells (APCs) involved in the T cell mediated immune response. We found that the B cell population was significantly decreased (P-value < 0.00001) in the COVID-19 patients compared to the healthy individuals. Interestingly, recovered patients had a higher number of these cells, although not similar to the healthy. However, the DC population increased in the positive patients which increased further in the recovered individuals. On the other hand, we observed a decrease in the CD8+ T cells and increase in the CD4+ T cell population in the COVID-19 patients. We also observed an increased abundance of activated CD4+ T cells compared to the Naïve CD4+/CD8+ T cells in the COVID-19 patients. Together, these indicate a suboptimal MHC Class I mediated but increased MHC Class II mediated antigen presentation in the COVID-19 patients. On the other hand, the increased abundance of the APCs and the CD8+ T cells in the recovered, possibly indicate recovery of the loss of MHC Class I mediated antigen presentation function.

### Increased cytotoxicity in response to COVID-19

The monocytes and NK cells confer the ‘killing function’ and cytotoxicity. We observed an increased killer cell population (Natural killer or NK and NK T cells) in the infected patients, which subsequently decreased in the recovered. The proliferating NK cells were the highest in recovered individuals, correlating with the half-life of the mature NK cells as well as the infection duration (28). The higher abundance of the proliferating NK cells in the recovered indicates the decrease in IFN-γ production post-infection (29). The classical monocyte population also followed a pattern like the NK cells, while the CD14+/CD16+ Intermediate monocyte, highly abundant in the healthy individuals, decreased significantly in the COVID-19 patients followed by an increase in the recovered individuals. This suggests higher activation of monocytes in the COVID-19 patients, followed by a partial restoration in the recovered individuals. Together, the increased ‘killer cell’ population and monocyte suggest an increased cytotoxicity in the COVID-19 patients. The statistical significance of the frequency of the cell types across three groups as well as pairwise comparison groups are available as **Supplementary File 1**; **Table S3**.

### Reduced immune response and increased inflammatory response by naïve CD4+/CD8+ T cells in the recovered individuals

The cell type specific expression of the surface markers and intracellular markers are presented in the **Figures 1D, E**. **Figure 1F** shows the expression of the cell type specific markers used for the cell type annotation across groups. We also looked at the cellular heterogeneity in a pairwise comparison (healthy vs COVID-19 positive, COVID-19 positive vs recovered, and healthy vs recovered) (**Figure 1G; H**, **Supplementary File 1**, **Figures S1B-1E**). Surprisingly, we found an unidentified cluster to be present explicitly in the healthy and was absent in the recovered (**Figure 1G, H**; **Supplementary File 1**; **Figure S1F**). Upon annotation using scpred, it was found to be the Naïve CD4+ T Cell population, with reduced expression of *MALAT1, BACH2* and several ribosomal protein coding genes when compared to the existing Naive CD4+/CD8+ T cells of the healthy/recovered individuals (**Figure 1I**; **Supplementary File 2**). Reduced expression of these genes confers increased immune response, reduced cytokine production and inflammatory response (**Figure 1J**) (30–32). Thus, loss of this Naïve CD4+ T cell population suggests a reduced immune response and increased inflammatory response by the Naïve CD4+/CD8+ T cells in the recovered individuals.

### Loss of B cell maturation process in the recovered individuals

To understand the cross-talk across cell types and subsequent regulation of immunological functions, we performed cell-cell communication analysis using the CellChat R package (33). The cell-cell communication analysis revealed a CD22-CD45 signaling between the Class-switched memory B cell and the Activated CD4+ T cells, in healthy but not in the COVID-19 positive or recovered (**Supplementary File 1**; **Figures S1G-1I**). It is important to note that the CD45, negatively regulates the CD22 level, which is crucial for B cell maturation and antibody production (34). Absence of CD22-CD45 interaction, thus indicates dysregulated B cell maturation in the COVID-19 patients. On the other side, CD99 signaling in the NK cells and CADM1 signaling in the CD8+ TCM were observed in the COVID-19 patients but not in healthy or recovered individuals. CD99 signaling in NK cells are known to upregulate the IL-6 and TNF-α (35), whereas the CADM1 signaling is known to increase the cytotoxicity and IFN-γ secretion (36). Together, these suggests a NK and CD8+ T cell-mediated elevated immune and inflammatory response in the COVID-19 patients.

### Compromised adaptive immunity in COVID-19 patients is mediated by CD40-CD40LG, enriched in the monocyte and T cells

**Figure 2:**
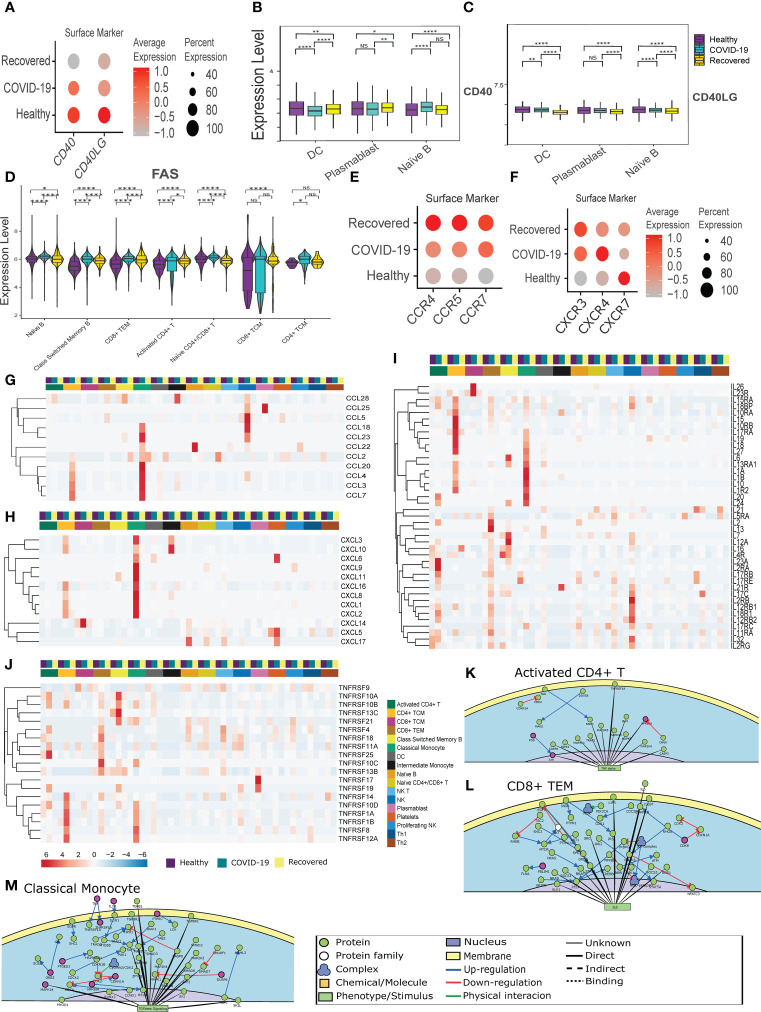
CD40-CD40LG mediated Inflammatory response in the COVID-19 patients. **(A)** CD40-CD40LG expression at surface level across the three groups of COVID-19 active infection, healthy and the recovered individuals. Color scale denotes the relative expression whereas circle size denotes the percentage of cells expressing the marker. **(B, C)** Cell- type specific surface expression of CD40-CD40LG across the 3 groups. **(D)** Surface level expression of FAS in the B and T cells. **(E, F)** Chemokine and Chemokine receptor expression at the surface level across the three groups. **(G–J)** RNA level expression of **(G)** Cytokines, **(H)** Chemokines, **(I)** Interleukins and **(J)** TNF Receptor Superfamily across all the cell types. **(K–M)** PPI level interaction network at **(K)** Activated CD4+ T cells, **(L)** CD8+ TCM, and **(M)** monocytes. [NS represents non-significant, *represents p-value < 0.05, **represents p-value < 0.01, **** represents p-value < 0.0001].

The immune and inflammatory response in the COVID-19 patients, as revealed by the cell-cell communication analysis, is modulated not only by CD22-CD45 interaction, but also CD40 and CD40LG interaction. However, the CD40-CD40LG interaction was not observed in our cell-cell communication analysis. The CD40-CD40 ligand expression and interaction are important not only for the activation of adaptive immune response, but also infection induced inflammation and CD8+ T cell apoptosis (37). Therefore, we looked at the CD40-CD40LG expression between the groups. **Figure 2A** shows the surface level expression of CD40-CD40LG, wherein we observed a decreased expression of CD40-CD40LG in the COVID-19 patients, with the further decreased expression in the recovered individuals, especially in the professional antigen presenting cells (APC) (**Figures 2B, C**). This indicates a decreased adaptive immune response during COVID-19, increased infection induced inflammation and CD8+ T cell apoptosis.

Subsequently, we looked at the expression of the FAS receptor, a death receptor expressed during apoptosis. We observed an increased expression in all the CD4+ and CD8+ T cell populations. Surprisingly, we also observed a higher expression of *FAS* in the B cell population (Naïve B cell, Class-switched Memory B cells), indicating an abnormal antigen presentation and antibody response in the COVID-19 patients (**Figure 2D**). Further, chemokine and cytokine receptors (*CXCR3, CXCR4, CXCR5, CCR4, CCR5*, and *CCR7*) expression was increased in active COVID-19 and recovered patients (**Figures 2E, F**). Alongside, we observed a high expression of cytokines (*CCL3, CCL4, CCL5, CCL7, CCL18* and *CCL20*) in the CD4+ TCM, Classical monocytes and NK cells in the COVID-19 patients (**Figure 2G**). A higher expression of chemokines (*CXCL1, CXCL2, CXCL3, CXCL8* and *CXCL16*) were also found in the CD4+ TCM and Classical Monocytes in the COVID-19 patients (**Figure 2H**). This indicates a CD4+ TCM and monocyte driven increased cytokines and chemokines in the active COVID-19 patients. Also, the interleukins (*IL1B, IL15, IL16*, and *IL32*) were found to be upregulated in the active COVID-19 patients, indicating a T cell and monocyte-mediated proinflammatory response during active COVID-19 (**Figure 2I**).

We observed increased expression of *TNFRSF1B*, also known as TNF receptor 2 (*TNFR2*) in the Classical monocytes and NK cells during active COVID-19 (**Figure 2J**). *TNFR2* is involved in the regulation of inflammation in the macrophage and CD8+ T cells. *TNFRSF13C*, a pro-survival receptor for B cells, was found to have increased expressed in the Class-switched memory B cells during active COVID-19 (38). Deficiency of *TNFRSF13C* is characterized by low circulating B cells, serum IgG and IgM but high levels of IgA (39). Thus, the very low expression of *TNFRSF13C* in the Class-switched memory B cells in the recovered individuals lends support to our earlier finding of decreased Class-switched memory B cells in the recovered individuals. It also indicates increased IgA antibody, and not IgG or IgM antibody in the recovered. The increased expression of *TNFRSF14* and *TNFRSF17* in the recovered individuals indicate an increased inflammatory response (**Figure 2J**). Together, the dysregulation of the CD40-CD40LG caused an increased monocyte and T cell mediated inflammatory response during active COVID-19. The overall differential expression of genes in activated CD4+ T cell, CD8+ TEM, Classical monocytes, NK cells, CD4+ TCM and CD8+ TCM are represented in the **Supplementary File 1**, 2; **Figures S2A-2F**. The protein-protein interaction-level network obtained from the differentially expressed genes revealed elevated TNF-α signaling in the Activated CD4+ T cells, IL-6 signaling in the CD8+ TEM cells, which are indicative of elevated inflammatory response during active COVID-19 (**Figures 2K, L**; **Supplementary File 1**, **Figure S2G**).

Surprisingly, we observed increased TGF-β signaling in the monocytes and NK cells (**Figure 2M**; **Supplementary File 1**; **Figure S2H**), indicating a pro-viral role of these cells (40). Besides, we also observed a decreased immune response and T cell activation in the CD4+ TCM and CD8+ TCM respectively, indicating an abnormal immune response and T cell activation (**Supplementary File 1**; **Figures S2I, J**). Besides, these also indicate the dysregulation of antigen presentation in the COVID-19 patients. Together, the results indicate a CD40-CD40 ligand interaction mediated increase of the cytokine response and abnormal T cell activation in the COVID-19 positive individuals.

### Immune, stress and antiviral responses are mediated by the T cells, monocytes and NK cells

**Figure 3:**
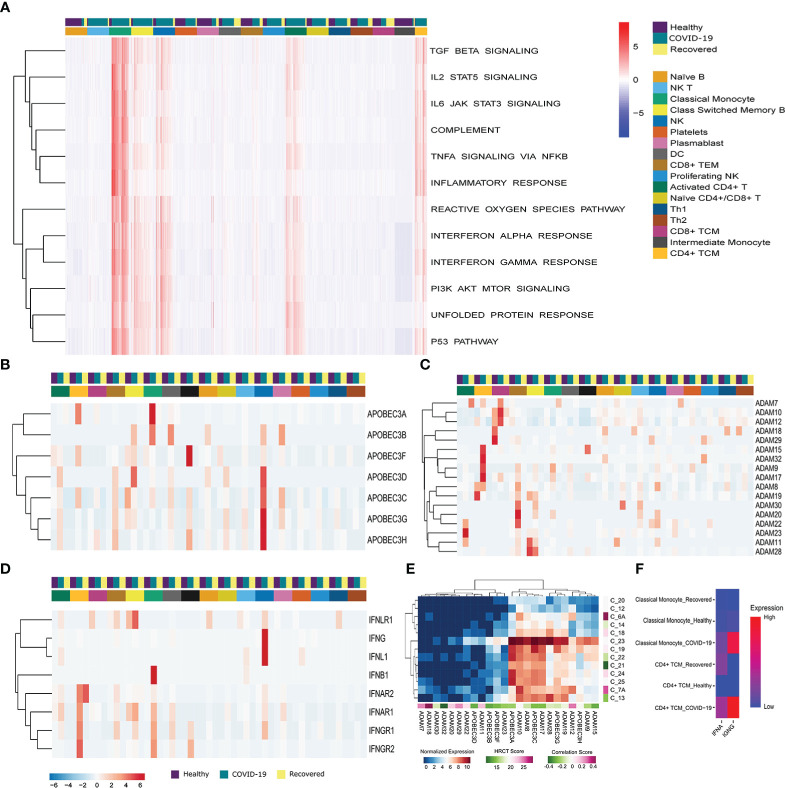
Immune, stress and antiviral Response during the SARS-CoV-2 infection. **(A)** G SEA at the single cell resolution across the three groups (COVID-19 patients, healthy and recovered), the row dendrograms distinguish the immune and stress response pathways. The cell types and the groups were highlighted using different color bars. Data is expressed as a relative enrichment score for each pathway. **(B, C)** Expression of **(B)** *ADAM*, and **(C)** *APOBEC3* genes. **(D)** Expression of antiviral genes in the COVID-19 patients and correlation with HRCT score. **(E)** Expression of IFN family genes across all the cell types across the three groups. Cell types and groups were highlighted using different color bars. **(F)** Cumulative expression of type I and type II IFN receptors between the Classical monocyte and CD4+ TCM across the three groups.

To understand the cell type specific immune and stress response dynamics, we performed GSEA at single cell resolution using Escape R/Bioconductor package by using molecular signature database (MSigDB-H) (**Figure 3A**) (41). We found immune response related pathways (TGF-β, IL2-STAT5, IL6-JAK-STAT3, and TNFα signaling, Complement system activation, and Inflammatory response pathway) to be upregulated in the classical monocyte, NK cells, activated CD4+ T cells and CD4+ central memory T cells in active COVID-19 patients. TGF-β and IL2 STAT5 signaling, as well as Complement system activation pathways were also upregulated in the Class-switched memory B cells during active COVID-19. On the other hand, the stress response related pathways (ROS pathway, Interferon alpha and gamma response, PI3K–AKT-mTOR signaling, UPR and p53 pathways) were also particularly enriched in the Classical monocyte, NK cells, activated CD4+ T cells, Class-switched memory B cells and CD4+ central memory T cells of the COVID-19 positive patients. Besides, the reactive oxygen species (ROS) and unfolded protein response (UPR) pathway, two major stress response pathways, were also enriched in the plasmablast in the recovered individuals. Together, these indicates T Cells, monocytes and NK cells mediated upregulation of the immune and stress response during active COVID-19.

To understand the dynamics of the antiviral response, we looked at the expression of *ADAM, APOBEC3* and *IFN* family members. We found a high expression of *APOBEC3A, APOBEC3B, APOBEC3C, APOBEC3D, APOBEC3F, APOBEC3G* and *APOBEC3H* in the classical, intermediate monocytes and NK cells of the COVID-19 patients compared to the healthy or recovered individuals (**Figure 3B**). On the other hand, ADAM family members were upregulated in the T cells (Activated CD4+ T cells, CD4+ TCM, CD8+ TCM, CD8+ TEM) in active COVID-19 compared to the healthy and recovered (**Figure 3C**). This highlights a T cell, monocyte and NK cell mediated upregulation of antiviral response during the SARS-CoV-2 infection. Within our data, we also checked for the possible association of the increased antiviral response with the disease severity within the COVID-19 positive patients. We found that the expression of the antiviral genes negatively correlated (except *ADAM7, ADAM11, ADAM12, ADAM18, ADAM20*, and *ADAM29*) with the HRCT score of the COVID-19 patients suggesting association of increased antiviral response with decreased disease severity (**Figure 3D**).

Finally, we found *IFNAR1* and *IFNAR2*, a receptor for type I interferon (IFNα) to be upregulated in the CD4+ TCM and classical monocytes of the COVID-19 patients, compared to the others. Receptors for type II interferon, *IFNGR1* and *IFNGR2* were also upregulated in the CD4+ TCM and classical monocyte (**Figure 3E**). However, the expression of type II IFN receptors was higher in COVID-19 patients in both the cell types, whereas type I IFN receptors were upregulated in the CD4+ central memory T cells of the recovered individuals (**Figure 3F**). In summary, inferences from our data suggests that the antiviral response is mediated by the central memory T cells, monocytes and NK cells during the SARS-CoV-2 infection and transcription of proinflammatory cytokines are increased as a result of type II IFN receptor mediated signaling during the SARS-CoV-2 infection.

### Aberrant activation of T cells in COVID-19 positive individuals

**Figure 4:**
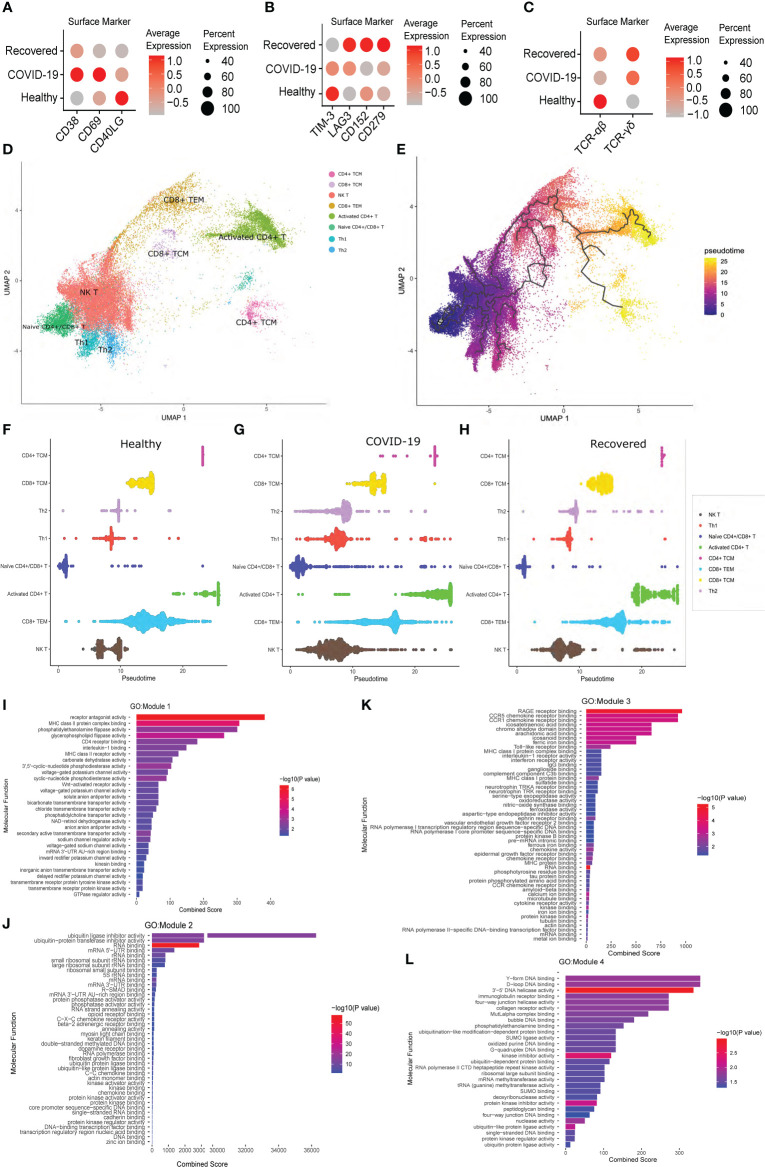
T Cell specific surface marker expression and Pseudotime analysis across the healthy, COVID-19 positive and recovered individuals. **(A)** Expression of the T cell activation markers at the surface marker level. **(B)** Expression of T cell exhaustion markers at the surface marker level. **(C)** Expression of TCR αβ and γδ chain at the surface marker level. Color scale denotes the relative expression whereas circle size denotes the percentage of cells expressing the marker. **(D)** UMAP visualization of T cell subtypes. **(E)** UMAP visualization of T cell subtypes with respect to pseudotime. **(F–H)** Distribution of cells against pseudotime for T cell subtypes across **(F)** healthy, **(G)** COVID-19 positive, and **(H)** recovered individuals. **(I–L)** GO enrichment of genes from **(I)** Module 1, **(J)** Module 2, **(K)** Module 3, and **(L)** Module 4.

To understand the T cell dynamics across the three groups, we looked for the expression of the T cell activation and exhaustion markers. We observed higher expression of CD38, CD69 and CD40LG during active COVID-19 infection, indicating an activated phase of T cell population, compared to the healthy and the recovered, where the T cells are at resting phase in comparison to the active infection (**Figure 4A**). We also observed higher expression of T cell exhaustion markers, i.e., *TIM-3, LAG3, CD152* or *CTLA4* and *CD279* or *PD-1* in the recovered individuals compared to the active COVID-19 and healthy individuals (**Figure 4B**). *This indicates that a larger population of the T cells are exhausted post recovery from the COVID-19*.

We then looked at the expression of a specific T cell receptor type. Generally, around 95% of the T cell receptor carry αβ chain, and T cells carrying αβ receptor chain respond to the pathogen in an antigen specific manner to induce cytokine productions followed by B cell maturation and antibody secretion. However, a small fraction of T cells carrying γδ receptor chains are essential in the initial immune and inflammatory responses (42). Surprisingly, we observed a gradual shift of TCR from αβ to γδ type across healthy, active COVID-19 and recovered, with highest expression of TCR γδ in the recovered (**Figure 4C**). Thus, the T cell exhaustion and the abundance of TCR γδ in the recovered individuals indicate an abnormal T cell response during the active COVID-19.

To understand the difference of T cell subtypes across the three groups, we constructed cell trajectory with respect to pseudotime using Monocle 3 (**Figures 4D, E**) (43). The cells for each cell type were plotted against the pseudotime for all the three groups and a significant difference of median pseudotime was observed (**Figures 4F–H**; **Supplementary File 1**; **Figure S3**). Importantly, we observed a lower pseudotime in the COVID-19 patients (except for the CD8+ TEM and CD4+ TCM), possibly indicating a faster transition from one cell type and/state to another. Finally, to understand the co-expression of genes with respect to pseudotime, we clustered the genes that were differentially expressed across healthy, COVID-19 positive and recovered, in each T cell subtypes, and identified four modules of co-expressed genes. While module 1, 2 and 4 showed significant GO enrichment (p-value <0.05) for the housekeeping cellular functions required for cell growth and development, module 3 showed significant enrichment for the immune and inflammatory response associated molecular functions (**Figures 4I–L**; **Supplementary File 4**). This indicates the possible impact of COVID-19 on normal cell functions beyond the conventional immune/inflammatory response.

### Distinct immune response signatures as revealed by similarity network fusion and machine learning model

**Figure 5:**
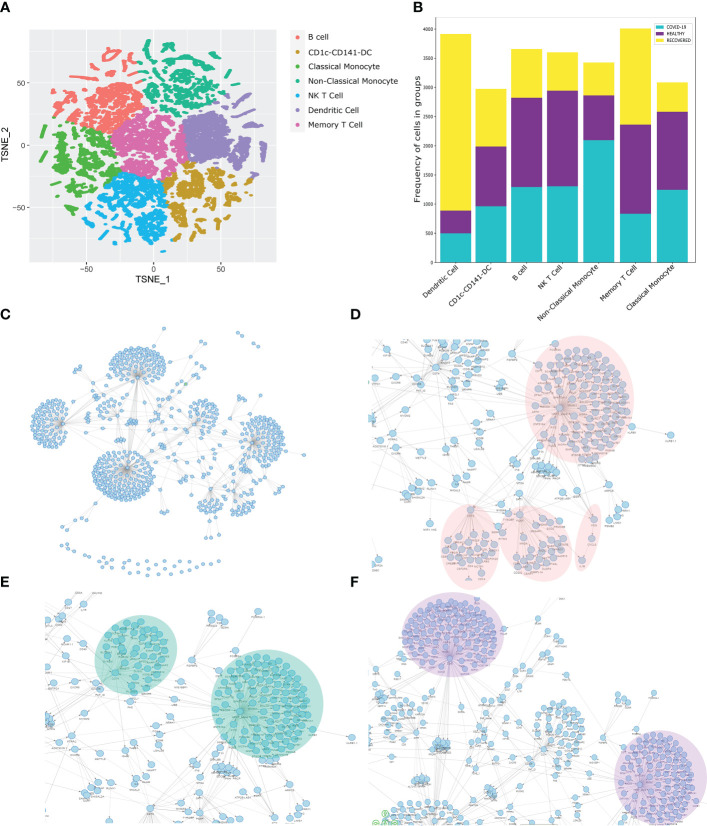
Similarity Network Fusion and Bayesian Network Model for Biomarker discovery. **(A)** SNF clustering of cells based on the gene expression, surface marker expression and clinical details of the individuals. **(B)** Distribution of the identified cell types across the healthy, COVID-19 positive and recovered individuals. **(C)** Bayesian Network Model built on the SNF clusters, gene expression, surface marker expression, and clinical data including the HRCT score of the individuals. **(D–F)** Specific highlights from the Bayesian Network Model showing the association of HRCT score with **(D)** *FOS, CST3, PSAP*, **(E)** *CD74*, and **(F)** *CD45*.

To understand the multiple modalities of the data and their association with the COVID-19 disease, we integrated gene expression, surface marker expression and clinical details of the individuals to perform a similarity network fusion (SNF)-based clustering (**Figure 5A**). The Dendritic cell population was found to be decreased in the COVID-19 patients and increased in the recovered individuals, reiterating our initial results towards a dysregulated antigen presentation in the infected individuals, followed by recovery of the function, post-infection (**Figure 5B**). We also observed a decrease in the NK T cells and monocyte populations post-infection, highlighting a reduced cytotoxicity in the recovered individuals. Besides, the upregulation of *S100A8*, *TXNIP* and *MT2A* genes in the monocytes indicate an inflammatory response upregulation in the infected individuals. We also observed an increased CD69+ memory T cell population in the recovered individuals. High expression of CD69 in the memory T cell population indicates higher tissue residence of the memory T cell population in the recovered individuals. Together, these results highlight a dysregulated antigen presentation and increased cytotoxicity in the infected individuals, and their reversal post-infection.

Towards the quest for the biomarkers associated with COVID-19 in our study cohort, we build a Bayesian Network model (**Figure 5C**). The complete network is available at Zenodo (https://doi.org/10.5281/zenodo.6583269). Importantly, we observed a strong connection between the HRCT score, *FOS* and *CXCL8* genes (**Figure 5D**). *FOS* is known to regulate cell death, apoptosis and inflammatory response, (44) which is one of the most significantly differentially expressed genes in the MERS and SARS-CoV-2 infection. The *CXCL8* is a neutrophil chemoattractant which recruits the neutrophil at the site of infection and initiates a proinflammatory response mediated by the *IL-8* and other cytokines (45). We also observed a direct association of *IL1β* with the *CXCL8*. Elevated *IL1β* and *IL6* activity is a feature of COVID-19 disease and dysregulation of the same is associated with disease severity (46). Thus, the association of *FOS, CXCL8* and *IL1β* with HRCT score make them a strong predictor of COVID-19 disease. Besides, we identified a strong association of HRCT score with *CST3*, a marker gene for monocytes. Monocytes, as we have shown earlier, are involved in the inflammatory responses in COVID-19 patients. Besides, the association of CST3 with *S100A9* also indicates a correlation between monocyte-mediated inflammatory response and HRCT score, which supports our previous findings. We also found a strong association between HRCT score and *PSAP*, a gene involved in antigen presentation and male fertility, both are perturbed in severe COVID-19 patients (47, 48).

We observed a strong association of HRCT score with *CD74*, also known as MHC Class-II invariant chain (**Figure 5E**). *CD74* is involved in MHC Class-II mediated antigen presentation, a process upregulated in the COVID-19 patients. Besides, *CD74* is known to have increased expressed in the severe COVID-19 patients (49). *CD74* also has immune-suppressant role, often observed in severe COVID-19 patients (50). Finally, we observed a strong association of *CD45* with HRCT score though *PDE11A* (**Figure 5F**). *PDE11A* is known to be involved in inflammatory response and is abundantly expressed in the severe COVID-19 patients (51). Just like *CD74, CD45* is also an immune-suppressant, and therefore enhanced expression in the severe COVID-19 patients. Therefore, the association of *CD74* and *CD45* with the HRCT score suggests the possible role of *CD74* and *CD45* in the COVID-19 disease and can possibly be used as a biomarker for the disease.

## Discussion

**Figure 6:**
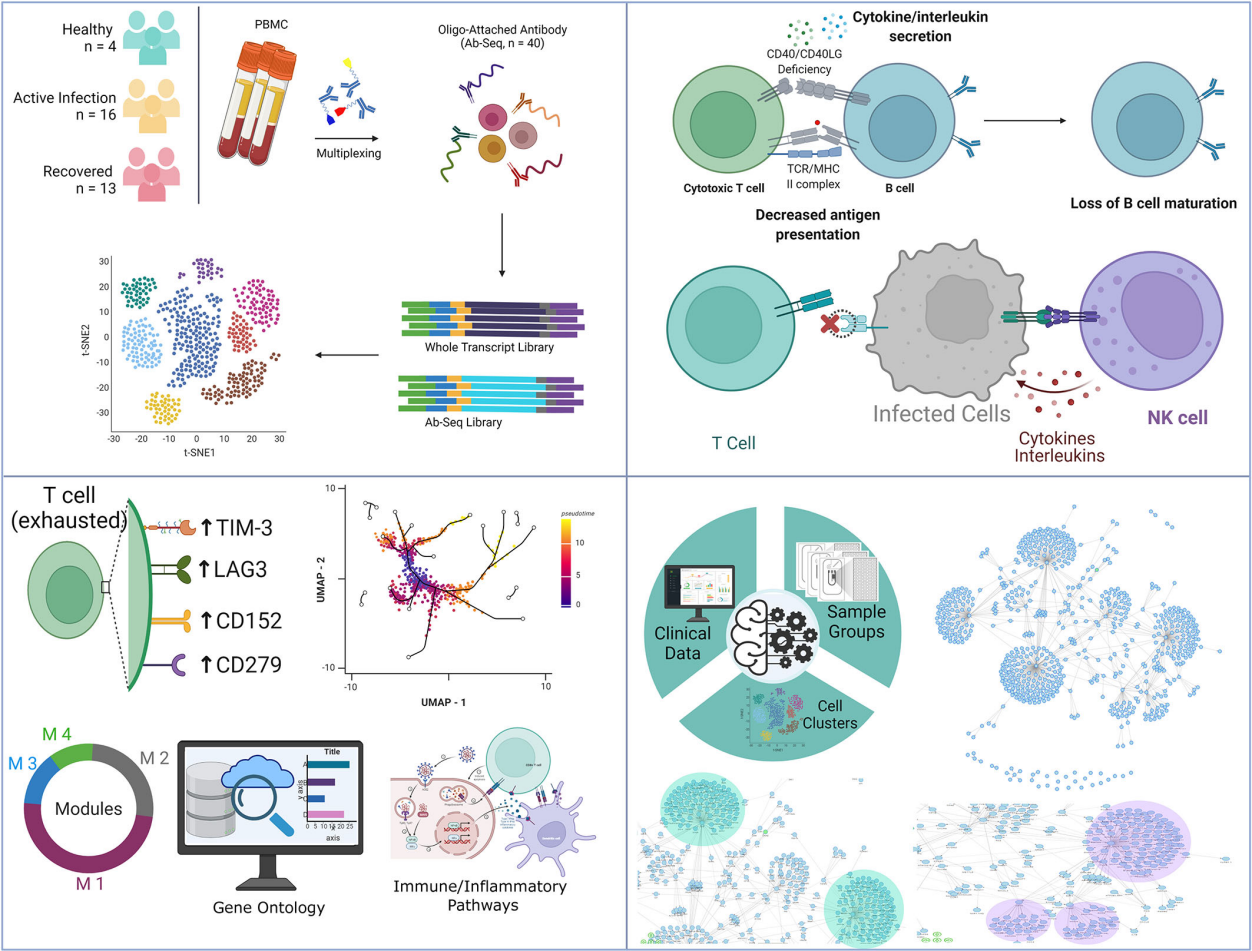
Summary of the key findings from the study. It highlights the observed T-cell dynamics within the COVID-19 patients, recovered individuals and healthy, as well as key immune findings harnessing strength of machine learning.

Although several studies have revealed the dysregulation of the immune system during COVID-19 disease (52, 53), there are still a few critical missing links in our understanding of the mechanisms behind the immune response dysfunction. Using single-cell resolution transcriptomics and targeted proteomics of 33 individuals (healthy, active COVID-19, and recovered), this study highlights major shifts in immune regulation occurring in the COVID-19 disease. While the decrease in B cell and CD8+ T cell population suggest a decreased MHC Class I mediated antigen presentation, the slight increase of DC and CD4+ T cell populations indicate an elevated MHC Class II mediated antigen presentation in the COVID-19 positive individuals. However, the decreased immune response by the CD4+ T cells of the infected individuals, as revealed by the PPI network, highlights suboptimal MHC Class II mediated antigen presentation. The high expression of FAS on the surface of B cells highlights B cell senescence in active COVID-19 cases, further strengthening our finding of diminished MHC Class I mediated antigen presentation. Increase in the B cell and CD8+ T cell population in the recovered group highlights the restoration of the MHC Class I mediated antigen presentation function. While the NK cell and classical monocyte population, the master regulator of the cytokine and chemokine response, were increased in the infected individuals followed by a decrease in the abundance in the recovered individuals, it was interesting to observe the increased abundance of proliferating NK cells in the recovered individuals (**Figure 6**).

Although not a well-studied cell population, the high abundance of proliferating NK cells in the recovered individuals can be explained with the 7-10 days half-life of the mature/activated NK cells, which then requires proliferation to maintain the desired level (28). NK cell activation is a distinct feature of COVID-19 and the average duration of the infection coincides with the half-life of activated NK cells (54), highlighting the process of restoration of the NK cell pool, post-infection. Besides, proliferating NK cells mediated reversal of the IFN-γ production (55), which increases during active infection, highlights the significance of this particular cell type. The Naïve T cell subset in the healthy individuals have higher immune response and decreased inflammatory response potential compared to the Naïve CD4+/CD8+ T cell population. Loss of this Naïve T cell subtype in the recovered individuals, thus highlights higher inflammatory response potential compared to the healthy. Cell-cell communication analysis reveals intra- and inter-cellular communication based on ligand-receptor interaction.

Our analysis revealed a shift in the intra- and inter- cellular communication between healthy to infected and to recovered individuals. While the CD22-CD45 signaling in the healthy group, involved in the B cell maturation process was absent in the infected and recovered group, the CD99 and CADM1 signaling observed in the infected group were absent post- infection. Thus, although the NK cell and CD8+ T cell mediated cytotoxicity were reversed in the recovered individuals, the B cell maturation process was still impaired in the recovered individuals, despite the increased abundance of B cells in the recovered individuals.

The CD40 and CD40 ligand interaction is known to regulate a wide range of immunological events, including infection-induced inflammatory response, CD8 T cell senescence and increased viral replication (56). The gradual decrease in the expression of CD40 and CD40LG in the COVID-19 positive and recovered groups suggest an enhanced infection-induced inflammation and apoptosis of CD8+ T cells during COVID-19 infection. Indeed, we observed high expression of FAS on CD8+ T cells and decreased abundance of CD8+ T cells in the infected patients. Interestingly, earlier studies also report the T cell depletion caused by the high levels of FAS molecules in the severe COVID-19 disease (57). Disrupted antigen presentation and antibody response were also observed during COVID-19 from an increased expression of FAS receptors (pro-apoptotic molecules) in CD4+ T and CD8+ T cell populations as well as B cell populations (Naïve B cell, class-switched memory B cell) (58).

On the other hand, the chemokines, cytokines, and interleukins were upregulated in the infected individuals, wherein the upregulation was mediated by the CD4+ T cells, CD8+ T cells, NK cells and monocytes. Interestingly, these inflammatory markers were found to be downregulated in the recovered individuals, highlighting the importance of understanding the post infection phase. The cell type specific PPI network revealed elevated TGF-β signaling in the monocyte and NK cells. The TGF-β signaling acts as a double edge sword, wherein on one hand it activates CD8+ T cells, and on other side, it facilitates viral replication, thus revealing another aspect of monocyte and NK cell mediated modulation of COVID-19. Further, this also reiterates the decreased CD40-CD40 ligand interaction-mediated increase of viral replication in the infected patients.

We found several immune responses associated signaling pathways such as IL6-JAK-STAT3, IL2-STAT5, TGF-β, TNFα, Inflammatory response pathway, and Complement system activation, upregulated in the classical monocyte, NK cells, activated CD4+ T cells, and CD4+ central memory T cells during active COVID-19. Other pathways involved in the stress response such as ROS pathway, Interferon alpha and gamma response, PI3K AKT mTOR signaling, UPR pathway and p53 pathways were also elevated in classical monocyte, NK cells, activated CD4+ T cells, Class-switched memory B cells and CD4+ central memory T cells in the active COVID-19 patients. These findings suggest the upregulation of immune pathways and stress pathways mediated *via* T cells, monocytes, and NK cells and fall in concurrence with the previous studies where multiple immune response pathways activate in the COVID-19 patients (59, 60).

The antiviral response during active COVID-19 disease was confirmed with a high expression of APOBEC3 and ADAM family members in the classical monocytes, NK cells, and T cells, respectively. Both APOBEC3 and ADAM family members are known to confer innate immunity (61, 62) and therefore can possibly be responsible for enhancing the antiviral response in active COVID-19 through T cell, NK cell, and monocytes. The upregulated expression of type II interferon receptors (IFNAR1 and IFNAR2) in CD4+ TCM and classical monocytes were more pronounced in active COVID-19 groups compared to recovered, where type I interferon receptor (*IFNGR1* and *IFNGR2*) expression dominated, thereby suggesting a surge of proinflammatory cytokines mediated *via* type II IFN receptor signaling during the active COVID-19 disease.

Previous studies have reported the dysregulation of T cells during COVID-19 disease (63, 64) which was observed in this study as well. This is particularly important, since lymphopenia is a key feature of COVID-19 disease, and an altered lymphopenia is also observed in convalescent COVID-19 patients (65–67). A sharp decrease in the CD4+ and CD8+ T cell and a delayed T cell response were associated with higher COVID-19severity, and similar to our findings, a restoration of the same in the recovered individuals has been highlighted by limited yet important study (65). Several studies have also highlighted abnormal abundance of helper T cells (Th1/Th2/Th17) and its association with decreased viral clearance and higher disease severity during COVID-19 (68, 69). The lymphopenia observed in the COVID-19 patients is also characterized by the abnormal activation and exhaustion of the T cell. We found a significant increase in the expression of CD38 and CD69 in the active COVID-19 patients compared to the healthy and recovered, suggesting an activated T cell response. A high level of exhaustion markers has been reported in the severe COVID-19 patients (70, 71), however, we observed elevated expression of the T cell exhaustion markers like *LAG3, CTLA4*, and *PD-1* in the recovered individuals. Together, this reveals an active phase of T cells during COVID-19 disease where after recovery the exhausted population of T cells becomes dominant. We further investigated the T cell receptor (TCR) type, where a gradual shift of TCR from αβ to γδ type was observed, with maximum expression in the recovered individuals. As the γδ TCR is responsible for inducing initial immune and inflammatory responses against specific antigens (72), their presence in the recovered individuals reflects aberrant and divergent T cell dynamics during the COVID-19 disease. Through our pseudotime analysis, different cell trajectories for T cell subsets revealed a reduced pseudotime amongst the COVID-19 patients, thereby suggesting a rapid transition between T cell subtypes that might be responsible for inducing aberrant T cell activation. We also observed differences among the constructed modules of co-expressed genes across the healthy, COVID-19 positive, and recovered groups in the T cell subsets where out of 4 modules, 3 of them exhibited significant housekeeping cellular functions that possibly indicate varied consequences of COVID-19 disease.

Finally, our similarity network fusion-based clustering reiterates our findings of dysregulated antigen presentation in the COVID-19 patients, whereas reduced cytotoxicity and higher tissue residence of the memory T cells in the recovered individuals. Our Bayesian network model reveals *FOS, CXCL8* and *IL1β* as important predictors for HRCT scores. While the FOS gene is known to be associated with apoptosis (73), CXCL8-mediated recruitment and activation of neutrophils is responsible for causing pathogenesis of lower respiratory tract infection and if overproduced, leads to cystic fibrosis (74). It is also suspected of playing a role in endothelial dysfunction. SARS-CoV-2 has been known to induce the apoptosis pathway, which has been shown to be intricately connected with inflammation and fibrosis, leading to medical complications (75). Thus, the association of increased levels of *FOS, CXCL8* and *IL1β* in patients with higher HRCT scores in our study using the integrative model provides support to this finding. Furthermore, we also found a strong correlation between *CST3* and *S100A9* and simultaneously with HRCT score. The monocyte markers *CST3* and *S100A9* exhibit monocyte-mediated pro-inflammatory responses (76). Together their association with HRCT score suggests the involvement of monocytes in inflammatory response during the COVID-19 disease. HRCT score was also strongly associated with *CD74* and *CD45*. Both *CD74* and *CD45* are immune-suppressants and are known to be elevated in the severe COVID-19 patients. Therefore, their correlation with HRCT score possibly signifies their role in the COVID-19 disease progression and severity.

## Conclusion

Our Single cell based COVID-19 study, highlights a dysregulated antigen presentation, CD40-CD40LG deficiency-mediated heightened immune/inflammatory/stress and antiviral response in the COVID-19 positive and recovered individuals, faster cellular transition in the COVID-19 patients, and COVID-19 disease biomarkers such as *FOS, CD45*, and *CD74*. These findings may further assist in understanding the complexity of immune response heterogeneity that possibly can serve to delineate treatment strategies for SARS-CoV-2 infection. Besides, our study also highlights the importance of understanding the COVID-19 aftereffects in recovered individuals which may be relevant for the re-infection/s. Further, a follow-up study with longitudinal COVID-19 recovered individuals, potentially with differential disease severity, would further the understanding of immune response dynamics in the recovered individuals.

## Data availability statement

The data presented in the study are deposited in the NCBI GEO repository, accession number GSE201088.

## Ethics statement

The studies involving human participants were reviewed and approved by CSIR-IGIB’s Human Ethics Committee Clearance (Ref No: CSIR-IGIB/IHEC/2020-21/01). The patients/participants provided their written informed consent prior to participation in this study. The patients/participants provided their written informed consent to participate in this study.

## Author contributions

PC, formal analysis, investigation, data curation, visualization, and writing - original draft. KK, data curation and writing - original draft. MK, SW, and SS, investigation. PM, formal analysis, visualization, and writing - original draft. AA, formal analysis and writing - original draft. RM, RG, and AG, formal analysis. AY and PD, data curation. KT and MJ, resources. TS, formal analysis, visualization, writing - review and editing, supervision. RP, conceptualization, methodology, supervision, writing - review and editing, funding acquisition. All authors contributed to the article and approved the submitted version.

## Funding

This research was funded by Bill and Melinda Gates Foundation, [Grant number - INV-033578 and INV-030592].

## Acknowledgments

The authors duly acknowledge all the COVID-19 patients, healthy and recovered individuals who participated in the study. Authors also would like to acknowledge the support of D.Y. Patil Medical College, Hospital and Research Institute, Kolhapur, Maharashtra, India for providing the relevant samples for this study. Authors acknowledge the help and support from Dr. Aradhita Baral towards facilitation as Research manager and coordination with the funders, Dr. Mohd Faruq for experimental facilitation. Authors acknowledge the support of Anil Kumar and Nisha Rawat towards COVID-19 sample transport and sample management. PC, PD, KK and AY acknowledge the CSIR for their research fellowship.

## Conflict of interest

The authors declare that the research was conducted in the absence of any commercial or financial relationships that could be construed as a potential conflict of interest.

## Publisher’s note

All claims expressed in this article are solely those of the authors and do not necessarily represent those of their affiliated organizations, or those of the publisher, the editors and the reviewers. Any product that may be evaluated in this article, or claim that may be made by its manufacturer, is not guaranteed or endorsed by the publisher.

## Supplementary material

Supplementary File 1 Details of the oligo-attached antibodies (list and oligo sequences).

Supplementary File 2 Table showing differential gene expression between the new Naïve T cell subset vs Naïve CD4+/CD8+ T cell in the healthy vs recovered individuals.

Supplementary File 3 Pseudobulk differential gene expression of the cell types highlighted in the study.

Supplementary File 4 GO enrichment analysis of the genes from each module.
