# Supplementary material for: Single-cell multiomics revealed the dynamics of antigen presentation, immune response and T cell activation in the COVID-19 positive and recovered individuals
Source: Front Immunol. 2022 Dec 2;13:1034159. doi: 10.3389/fimmu.2022.1034159 (PMC9755500; doi:10.3389/fimmu.2022.1034159)
Supplement: Supplementary File 1 — Details of the oligo-attached antibodies (list and oligo sequences). [file DataSheet_1.pdf]

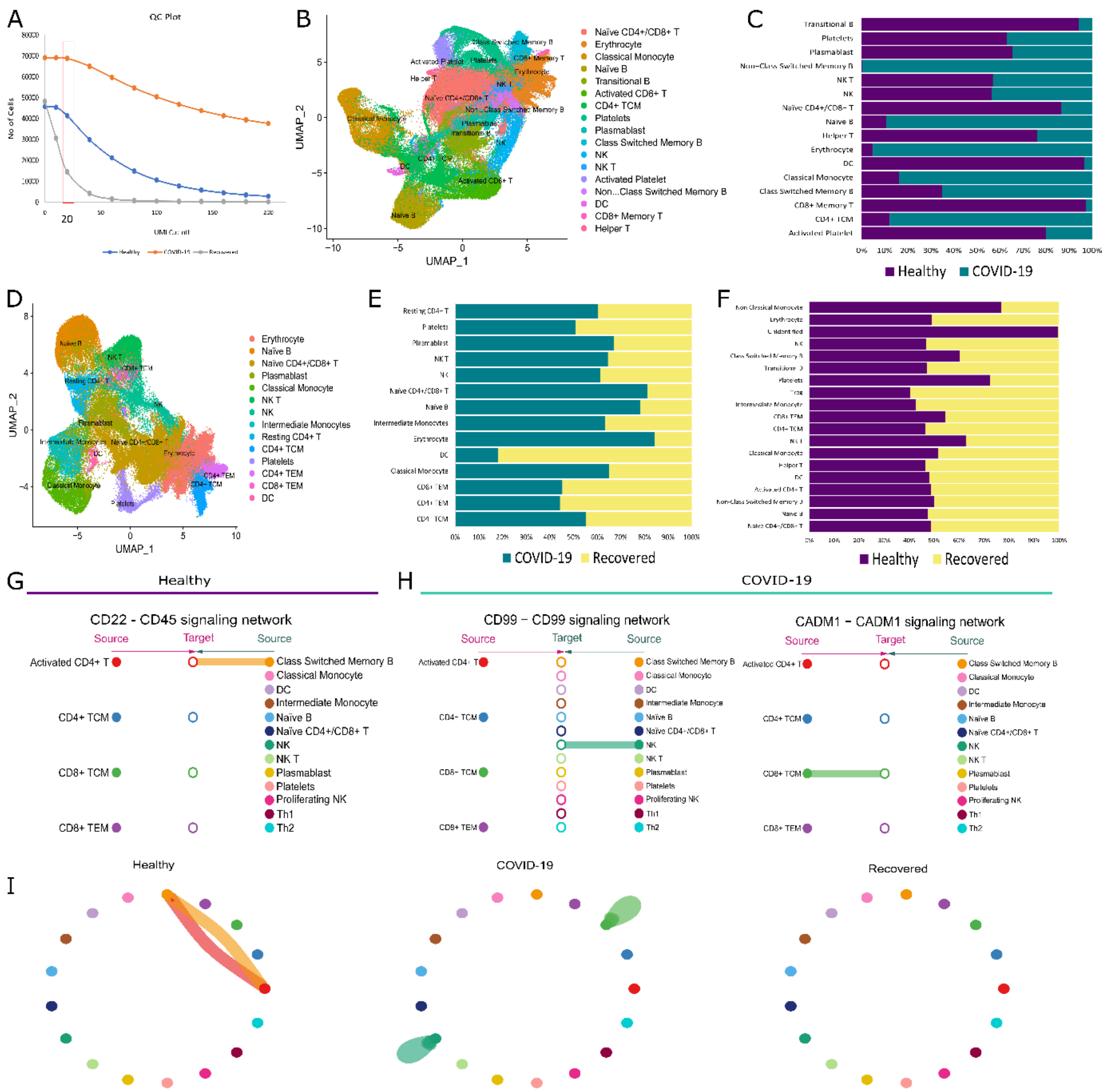

Supplementary Figure S1: Quality control, group-wise clustering of cells and cell-cell communication across Healthy, COVID-19 and Recovered individuals

(A) UMI cut-off plot showing number of unique UMI (x-axis) present in total number of cells (y-axis). Cut-off value of unique UMI count is highlighted. (B) Clustering and cell type annotation of Healthy vs COVID-19 positive group. (C) Distribution of cell types between Healthy and COVID-19 positive group. (D) Clustering and cell type annotation of COVID-19 positive vs Recovered group. (E) Distribution of cell types between COVID-19 positive and Recovered group. (F) Distribution of cell types between Healthy and Recovered group. (G-I)

Cell-cell communication analysis for (G) Healthy, (H) COVID-19 and (I) Healthy, COVID-19 and Recovered group combined.

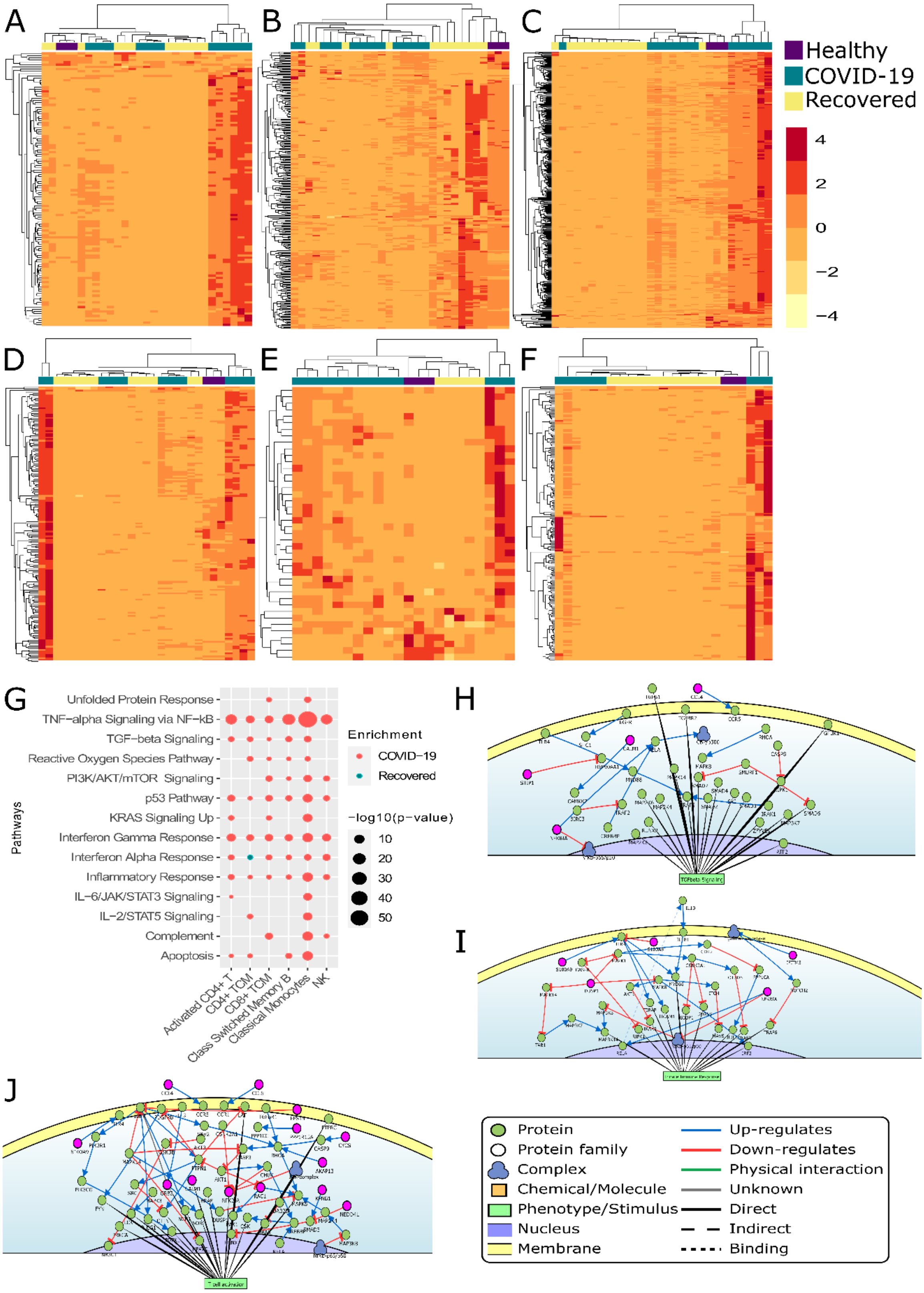

Supplementary Figure S2: Pseudobulk differential gene expression, pathway enrichment and cell level PPI across Healthy, COVID-19 and Recovered group

(A-F) Heatmap showing differential gene expression (A) Activated CD4+ T cell, (B) CD8+ TEM, (C) Classical monocytes, (D) NK cells, (E) CD4+ TCM and (F) CD8+ TCM. (G) Pathway enrichment analysis of differentially expressed genes. The size of the circles represents the significance of the enrichment score while colour represents the group where the pathway is enriched. (H-J) Cell level PPI of (H) NK cell, (I) CD4+ TCM and (J) CD8+ TCM

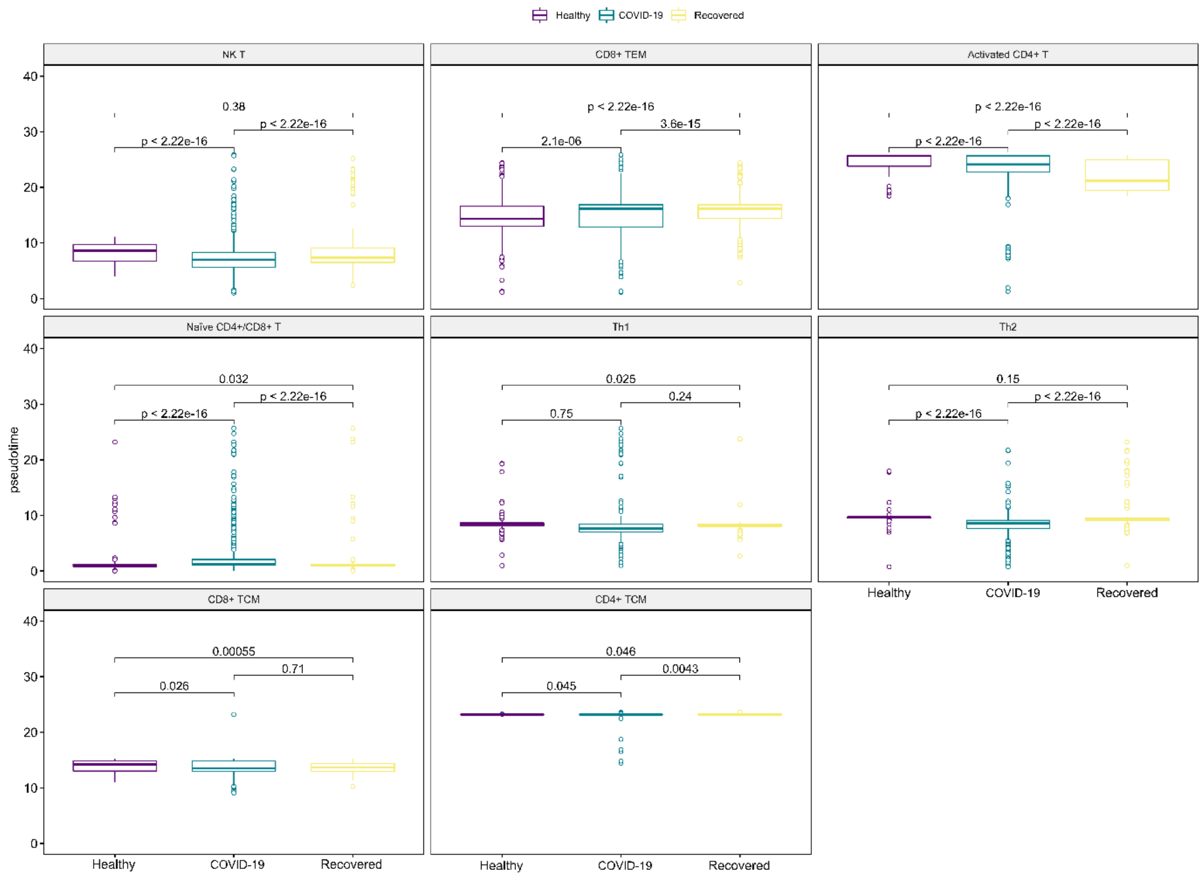

Supplementary Figure S3: Cell type-wise pseudotime for Healthy, COVID-19 and Recovered groups along with statistical significance (Kruskal-Wallis test) of the difference of median pseudotime.

Supplementary Table S1: List of surface marker-specific antibodies and attached oligo

Sl No	Surface Markers	Oligo sequence
1	CD195:2D7|CCR5|AHS0070	ATGGTTTAGTCGTACGTGGGTTTAGATTGGCGGTGC
2	HLA-DR|CD74|AHS0035	TGTTGGTTATTCGTTAGTGCATCCGTTTGGGCGTGG
3	CD40|CD40|AHS0117	GGTGTAATTGGGCTAGAACGTATATGCGGTAAGGCG
4	CD184|CXCR4|AHS0060	CAGTGTTTAGAGCGGGTTGCATATGTCGTTTAGAGG
5	CD49d|ITGA4|AHS0063	TAGGGTGACTTAGCGATTGATGCGTATGTTTGGGCG
6	TCR-alpha_beta|TRA_TRB|AHS0078	TTGCGTCGGATTATTAGTTCGGGTATTATGCGGTGC
7	CD152|CTLA4|AHS0017	TAGTATCCGTAGTAGTTATCTGCCCGTTCGTTATGC
8	CD95|FAS|AHS0023	GGCCCGTTAGAGTTGGTATCCGTATGAAGGTTAGCT
9	CD127|IL7R|AHS0028	AGTTATTAGGCTCGTAGGTATGTTTAGGTTATCGCG
10	CD25:2A3|IL2RA|AHS0026	AGTTGTATGGGTTAGCCGAGAGTAGTGCGTATGATT
11	CD62L:DREG-56|SELL|AHS0049	ATGGTAAATATGGGCGAATGCGGGTTGTGCTAAAGT
12	CD3:UCHT1|CD3E|AHS0231	AGCTAGGTGTTATCGGCAAGTTGTACGGTGAAGTCG
13	GITR|TNFRSF18|AHS0104	TCTGTGTGTCGGGTTGAATCGTAGTGAGTTAGCGTG
14	CD19:SJ25C1|CD19|AHS0030	TAGTAATGTGTTCGTAGCCGGTAATAATCTTCGTGG
15	CD194|CCR4|AHS0038	AATATTAGTGGGTCCTCGCGTTGGCCGGTTGTTAGT
16	CD161:DX12|KLRB1|AHS0002	GTTATGGTTGTCGGTAGAGTATCGTGTTGCGTTAGT
17	LAG-3|LAG3|AHS0018	CGGCATGAATTAGGCGAGACTTAGTATACGAGCTGG
18	CD183|CXCR3|AHS0031	AAAGTGTTGGCGTTATGTGTTCGTTAGCGGTGTGGG
19	CD39|ENTPD1|AHS0006	TGTTGTCTTGTACGGCTTGAGTCGGGTTAATTCGGG
20	CD45RA:HI100|PTPRC|AHS0009	AAGCGATTGCGAAGGGTTAGTCAGTACGTTATGTTG
21	CD7|CD7|AHS0043	GTATGTAGGTCTTATGTGTTGGCGTAGTATGCGTTT
22	CD16:3G8|FCGR3A|AHS0053	TAAATCTAATCGCGGTAACATAACGGTGGGTAAGGT
23	CD27:M-T271|CD27|AHS0025	TGTCCGGTTTAGCGAATTGGGTTGAGTCACGTAGGT
24	CD197|CCR7|AHS0007	AAGGGTTGTAAGTTAGTCGATCCGCGTATTGTCATG
25	CD154|CD40LG|AHS0077	TAAGAGGTAAGTGCATTCGGGTATAGGCGTGATTTG
26	CD294|PTGDR2|AHS0106	TTAGAGTTCGTGAGAGGGTAGATCGCGTTTGTAGCC
27	Tim3|HAVCR2|AHS0016	TAGGTAGTAGTCCCGTATATCCGATCCGTGTTGTTT
28	CD5:UCHT2|CD5|AHS0047	ACGAAGCGAGCGAAGAACCTATGCGATTGAGTAAGT
29	CD14:MPHIP9|CD14|AHS0037	TGGCCCGTGGTAGCGCAATGTGAGATCGTAATAAGT
30	CD8:RPA-T8|CD8A|AHS0027	TGATTGGGTACGCGCTTGGCTTATATAGTCGGGTCT
31	CD56:NCAM16.2|NCAM1|AHS0019	AGAGGTTGAGTCGTAATAATAATCGGAAGGCGTTGG
32	CD21:B-LY4|CR2|AHS0074	GTATTCGCGTATTGTCAGTCGGTAGGGTTATGGTCT
33	CD279:EH12-1|PDCD1|AHS0014	ATGGTAGTATCACGACGTAGTAGGGTAATTGGCAGT
34	CD45|PTPRC|AHS0040	GTGCGAAATGGCGGAATGTTATCTGCGAATGTAGTC
35	CD69|CD69|AHS0010	CAATAACGGGTCATAGTAAGTCGCGAGTAAGAGGGC
36	CD28:CD28.2|CD28|AHS0024	TTGGTTTCGTAAGCGGCTAAGCGTATCTCGTGTTTG
37	CD38:HIT2|CD38|AHS0022	GTCAACGATGGGTAGCGGTAGAAATAACGGAACTGG
38	TCR-gamma delta:B1|TRD_TRG|AHS0015	GATTCTTATAGTCGTTGCGTAGGTTCGTCTGTGAGT
39	CD4:SK3|CD4|AHS0032	TCGGTGTTATGAGTAGGTCGTCGTGCGGTTTGATGT
40	CXCR5|CXCR5|AHS0039	AGGAAGGTCGATTGTATAACGCGGCATTGTAACGGC

Supplementary Table S2: ROC, sensitivity and specificity of SVM-based annotation

Cell type	ROC	Sensitivity	Specificity
ASDC	0.996	0.987	0.988
B intermediate	0.998	0.962	0.992
B memory	0.999	0.997	0.997
B naive	0.999	0.984	0.998
CD14 Mono	0.999	0.996	0.992
CD16 Mono	0.999	0.982	0.995
CD4 CTL	0.995	0.91	0.987
CD4 Naive	0.998	0.993	0.977
CD4 Proliferating	0.999	0.963	0.997
CD4 TCM	0.988	0.913	0.973
CD4 TEM	0.978	0.826	0.963
CD8 Naive	0.998	0.97	0.994
CD8 Proliferating	0.998	0.989	0.996
CD8 TCM	0.979	0.809	0.973
CD8 TEM	0.995	0.973	0.978
cDC1	1	0.993	0.995
cDC2	0.999	0.986	0.997
dnT	0.985	0.68	0.994
Doublet	0.912	0.587	0.987
Eryth	1	0.965	0.999
gdT	0.993	0.907	0.986
HSPC	1	0.982	1
ILC	0.998	0.954	0.996
MAIT	0.999	0.986	0.992
NK	0.999	0.983	0.991
NK Proliferating	0.999	0.967	0.998
NK_CD56bright	0.999	0.998	0.996
pDC	1	0.999	1
Plasmablast	1	0.984	1
Platelet	0.988	0.945	0.998
Treg	0.997	0.906	0.994

Supplementary Table S3: The statistical significance of the frequency of the cell types across three groups as well as pairwise comparison groups

	Healthy	COVID-19	p-value	Healthy	Recovered	p-value	COVID-19	Recovered	p-value
**Activated CD4+ T**	358	4132	<0.00001	358	260	0.0013	4132	260	<0.00001
**CD4+ TCM**	15	523	<0.00001	15	15	1	523	15	<0.00001
**CD8+ TCM**	430	270	<0.00001	430	428	0.9621	270	428	<0.00001
**CD8+ TEM**	3762	2469	<0.00001	3762	1107	<0.00001	2469	1107	<0.00001
**Class Switched Memory B**	664	8970	<0.00001	664	305	<0.00001	8970	305	<0.00001
**Classical Monocyte**	1259	8894	<0.00001	1259	936	<0.00001	8894	936	<0.00001
**DC**	1042	4165	<0.00001	1042	2415	<0.00001	4165	2415	<0.00001
**Intermediate Monocyte**	814	59	<0.00001	814	60	<0.00001	59	60	1
**Naïve B**	18745	3176	<0.00001	18745	3488	<0.00001	3176	3488	0.0018
**Naïve CD4+/CD8+ T**	958	2572	<0.00001	958	535	<0.00001	2572	535	<0.00001
**NK**	1111	6588	<0.00001	1111	773	<0.00001	6588	773	<0.00001
**NK T**	837	15947	<0.00001	837	916	0.1282	15947	916	<0.00001
**Plasmablast**	5216	1528	<0.00001	5216	1151	<0.00001	1528	1151	<0.00001
**Platelets**	3829	3880	0.6453	3829	553	<0.00001	3880	553	<0.00001
**Proliferating NK**	944	3284	<0.00001	944	725	0	3284	725	<0.00001
**Th1**	734	1415	<0.00001	734	431	<0.00001	1415	431	<0.00001
**Th2**	642	951	<0.00001	642	445	<0.00001	951	445	<0.00001

Statistical significance was calculated using Fisher's exact test.
